# Electroencephalography for monitoring cortical electrical activity related to intracranial pressure in patients with traumatic brain injury: a systematic review

**DOI:** 10.3389/fneur.2026.1833935

**Published:** 2026-05-26

**Authors:** Jelmer-Joost Lenstra, Gustavo E. Marcolin, Bram Jacobs, Ulf Günther, Jan-Willem Elting, Gea Drost, Michel J. A. M. van Putten, Joukje van der Naalt, Harm J. van der Horn

**Affiliations:** 1 Department of Neurology, University of Groningen, University Medical Center Groningen, Groningen, Netherlands; 2 Department of Critical Care, University of Groningen, University Medical Center Groningen, Groningen, Netherlands; 3 Department of Clinical Neurophysiology, University of Groningen, University Medical Center Groningen, Groningen, Netherlands; 4 Department of Clinical Neurophysiology, Medisch Spectrum Twente, University of Twente, Twente, Netherlands

**Keywords:** electroencephalography, intracranial pressure, multimodal monitoring, systematic review, traumatic brain injury

## Abstract

Intracranial pressure (ICP) driven treatment of patients with traumatic brain injury (TBI) in the Intensive Care Unit (ICU) remains a continuous challenge. Patients are sedated and therapy focuses on preventing secondary injury caused by an increase in ICP and reduction of cerebral blood flow (CBF). While ICP-monitoring provides valuable information on secondary deterioration, it offers limited insight into the complex and dynamic intracranial processes following TBI and carries the risk of complications (hemorrhage, infections). Integration of noninvasive advanced monitoring modalities to understand this process is mandatory. Electroencephalography (EEG) monitoring provides an advanced noninvasive method to measure focal cerebral electrical activity in real-time and is particularly sensitive to cortical oxygen and metabolic deficits. Investigations of the relationship between disruptions in EEG patterns and traditional invasive ICP measurements could provide valuable insights into the pathophysiology of intracranial hypertension and may pave the way for using continuous EEG in future ICP management. To date, no systematic review has evaluated the relationship between EEG and ICP in adult patients with TBI. This systematic review was conducted according to the PRISMA-guidelines. Three studies specifically reported on the incidence of post-traumatic seizures and ICP, and one study reported on a directional relationship between EEG activity and ICP. Altogether, there is a paucity of evidence and studies investigating the relationship between EEG and ICP. This highlights the need for systematic, well-powered research to clarify their interplay and underlying pathophysiology.

## 1 Introduction

Moderate and severe traumatic brain injury (TBI) are characterized by high mortality and morbidity rates that place a substantial burden on patients, families, and healthcare systems worldwide (1). The direct impact to the brain sets off a complex chain reaction of cellular and biochemical processes that may eventually cause secondary brain edema and post-traumatic seizures. Management of patients with severe TBI in the Intensive Care Unit (ICU) primarily focuses on preventing and treating raised intracranial pressure (ICP) to optimize the cerebral perfusion pressure (CPP) (2). Monitoring of ICP is done using an invasive sensor, which is placed either intraventricular [which can be used as external ventricular drain (EVD)] or intraparenchymal. This device offers limited insight in dynamics of intracranial hypertension and further carries risk of complications (3). There is a need for more informative noninvasive tools for monitoring changes in ICP to prevent secondary deterioration, especially since early aggressive treatment of patients may improve outcome (4, 5).

Integration of electroencephalography (EEG) monitoring as noninvasive advanced monitoring modality may provide an opportunity to better understand intracranial dynamics, which is required to develop individualized care. Continuous EEG-monitoring is already recommended by the American Clinical Neurophysiology Society (ACNS) in critically ill patients to detect and treat seizure activity, identify cerebral ischemia, monitor sedation and (burst-) suppressive therapy, and assess severity and prognosis (6, 7). However, the relationship between EEG and ICP remains uncertain. An increase in ICP lowers CPP, which subsequently reduces cerebral blood flow (CBF), thereby limiting cerebral oxygen and metabolic demands. Under such challenging circumstances, the relative contribution of neurogenic mechanisms may predominate over vasomotor, chemical, and metabolic control of cerebral autoregulation (8). Continuous EEG may serve as an indirect metric for the course of ICP and may provide early signs of secondary deterioration as described in non-traumatic neurocritical patients (9, 10). Moreover, specific cortical rhythms reflect preserved neuronal function and may change when neuronal activity is compromised by oxygen and metabolic deficits due to changes in CPP and hypoperfusion. This systematic review evaluates the relationship between EEG changes and ICP in adult patients with moderate and severe TBI admitted to the ICU. It explores the potential role of EEG in multimodal monitoring for ICP management.

## 2 Methods

### 2.1 Search strategy

This systematic review was conducted in accordance with the Preferred Reporting Items for Systematic Reviews and Meta-Analyses (PRISMA) statement (11). A comprehensive literature search was performed across three electronic databases: PubMed, Embase, and Scopus. The final search was conducted on 26 September 2025 and went as far back as data were available. Three search strategies were used, consisting of index terms and their respective synonyms related to *(i)* brain injury, traumatic; *(ii)* electroencephalogram; and *(iii)* intracranial pressure. The complete search strategy for each database is provided in Table 1.

**Table 1:** Search strategies for PubMed, Embase, and Scopus.

PubMed 340 results	Embase 390 results	Scopus 143 results
(“Craniocerebral Trauma”[Mesh] OR “Craniocerebral Traum^*^”[tiab] OR “Traum^*^ Brain”[tiab] OR “Brain Traum^*^”[tiab] OR “Head Traum^*^”[tiab] OR “Brain Concussio^*^”[tiab] OR “Brain Contusio^*^”[tiab] OR “traum^*^ diffus^*^”[tiab] OR “traum^*^ focal”[tiab]) AND (“Electroencephalography”[Mesh] OR “Electroencephalogra^*^”[tiab] OR “EEG”[tiab]) AND (“Intracranial Pressure”[Mesh] OR “Intracranial Pressure^*^”[tiab] OR “Intracranial Hypertension”[Mesh] OR “Intracranial Hypertension”[tiab] OR “Intracranial Hypotension”[Mesh] OR “Intracranial Hypotension”[tiab])	(‘traumatic brain injury'/exp OR ‘brain concussion'/exp OR ‘brain contusion'/exp OR ‘Traum^*^ Brain':ti,ab,kw OR ‘Brain Traum^*^':ti,ab,kw OR ‘Head Traum^*^':ti,ab,kw OR ‘Brain Concussio^*^':ti,ab,kw OR ‘Brain Contusio^*^':ti,ab,kw OR ‘traum^*^ diffus^*^':ti,ab,kw OR ‘traum^*^ focal':ti,ab,kw) AND (‘electroencephalogram'/exp OR ‘Electroencephalogra^*^':ti,ab,kw OR ‘EEG':ti,ab,kw) AND (‘intracranial pressure'/exp OR ‘Intracranial Pressur^*^':ti,ab,kw OR ‘intracranial hypertension'/exp OR ‘intracranial hypertension':ti,ab,kw OR ‘intracranial hypotension'/exp OR ‘intracranial hypotension':ti,ab,kw)	INDEXTERMS (“Craniocerebral Trauma” OR “Brain Injuries, Traumatic”) OR TITLE-ABS-KEY({Traum^*^ Brain} OR {Brain Traum^*^} OR {Head Traum^*^} OR {Brain Concussio^*^} OR {Brain Contusio^*^} OR {traum^*^ diffus^*^} OR {traum^*^ focal}) AND INDEXTERMS (“Electroencephalography”) OR TITLE-ABS-KEY({Electroencephalogra^*^} OR {EEG}) AND INDEXTERMS (“Intracranial Pressure” OR “Intracranial Hypertension” OR “Intracranial Hypotension”) OR TITLE-ABS-KEY({Intracranial Pressure^*^} OR {Intracranial Hypertension} OR {Intracranial Hypotension})

### 2.2 Eligibility criteria

Articles fulfilling the following criteria were included for full text reviewing:

(I) *Population:* the study had to include patients who sustained a TBI with indication for ICP-monitoring. Studies based on pediatric patients (< 18 years) were not included in this review. Additionally, studies involving only patients with anesthetic-induced EEG burst suppression pattern as a second-tier strategy to reduce ICP were excluded, as this review focuses on detecting early signs of secondary deterioration. Studies with a combination of (in)eligible patients were individually considered by the reviewers (JJL and GEM).
(II) *Outcome:* the study had to report on the relationship (or lack thereof) between EEG and ICP. The EEG data needed to include specific modalities such as continuous electroencephalography (*cEEG*) or quantitative electroencephalography (*qEEG*). In line with the objective of this review, cerebral function monitoring (CFM) and invasive intracranial EEG data were not included. Subcutaneous EEG recordings were included. ICP-monitoring consisted of external ventricular drains (EVDs) or intraparenchymal probes.
(III) *Study design:* studies that did not present original data were excluded. Case reports and series were also excluded. Additionally, animal and *in vitro* models simulating TBI were excluded from this review.
(IV) *No full-text:* studies were excluded if the full manuscript was not available to the reviewers (i.e. only abstract, no accessibility) or if it was not written in English.

The hierarchy of exclusion was collectively decided as follows: *Study design (III), Population (I), Outcome (II), and No full-text (IV)*.

### 2.3 Study selection process

Two reviewers (JJL and GEM) independently screened titles and abstracts of all retrieved articles against the eligibility criteria. One reviewer (JJL) screened the reference lists of potentially relevant reviews to identify primary studies that may have been missed by the search. All eligible articles were then assessed through full-text screening (JJL and GEM). Discrepancies regarding selected articles were resolved through discussion (JJL, GEM, and HJvdH). The Rayyan web application was implemented for independent article screening (12).

### 2.4 Data extraction

Two reviewers (JJL and GEM) independently reviewed the relationship between EEG and ICP using extracted data including publication year, study design, and sample details. Consensus according data inclusion was reached through discussion (JJL, GEM, and HJvdH).

### 2.5 Quality assessment

The methodological quality of the included studies was independently assessed by two reviewers (JJL and GEM) using the Joanna Briggs Institute (JBI) critical appraisal checklist for cohort studies (Table 2) (13). The checklist consists of 11 questions scored as *Yes, No, Unclear*, or *Not applicable*. The total number of *Yes* responses classifying studies as: *poor (*<*4), moderate 4–7*, or *good (*≥*8)*. The methodological quality was not considered during study selection.

**Table 2:** Methodology assessment.

JBI checklist for cohort studies	Vespa et al. (14)	McNamara et al. (15)	Vespa et al. (16)	Sanz-García et al. (17)
1. Were the two groups similar and recruited from the same population?	Y	N	Y	N
2. Were the exposures measured similarly to assign people to both exposed and unexposed groups?	Y	Y	Y	Y
3. Was the exposure measured in a valid and reliable way?	Y	Y	Y	Y
4. Were confounding factors identified?	Y	N	Y	Y
5. Were strategies to deal with confounding factors stated?	Y	N	Y	Y
6. Were the groups/participants free of the outcome at the start of the study (or at the moment of exposure)?	Y	N	Y	Y
7. Were the outcomes measured in a valid and reliable way?	Y	U	Y	Y
8. Was the follow up time reported and sufficient to be long enough for outcomes to occur?	Y	Y	Y	Y
9. Was follow up complete, and if not, were the reasons to loss to follow up described and explored?	Y	Y	Y	
10. Were strategies to address incomplete follow up utilized?	N/A	N/A	N/A	N/A
11. Was appropriate statistical analysis used?	Y	N	Y	Y
Appraisal	Good 10/11	Moderate 4/11	Good 10/11	Good 9/11

## 3 Results

**Figure 1:**
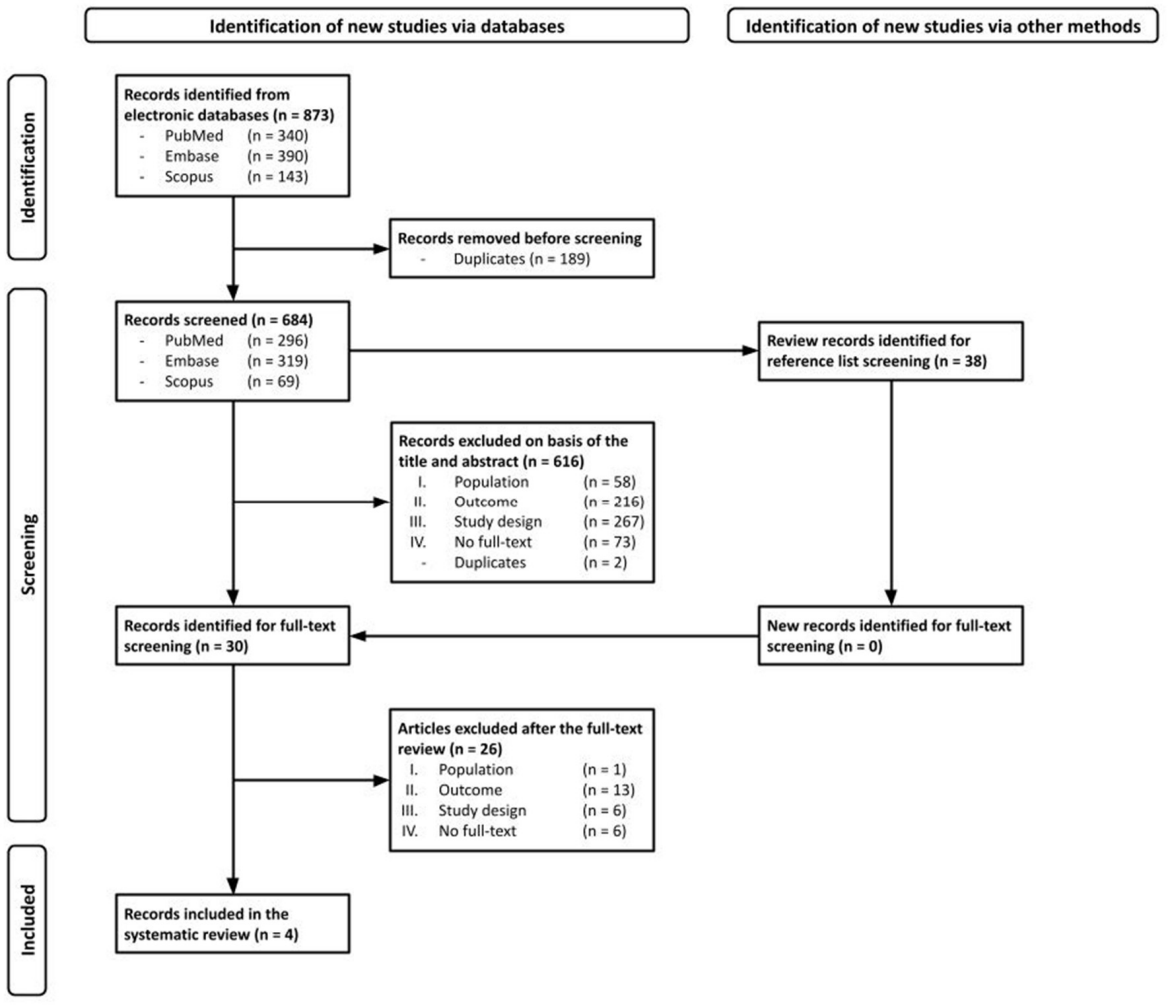
PRISMA flow diagram exhibiting the records selection process.

Our search yielded a total of 873 results, of which at first 189 were identified as duplicates and removed (Figure 1). Titles and abstracts of the remaining 684 articles were screened according to the eligibility criteria. A total of 30 articles were selected for full-text screening. In addition, reference lists of 38 review articles were screened for additional primary studies, which did not yield any studies that had not already been identified through the search strategy. Following the full-text review, consensus was reached to exclude 26 articles. Finally, four articles met our selection criteria and were included in the systematic review.

### 3.1 ICP and post-traumatic seizures

Vespa et al. examined the incidence and relationship between post-traumatic seizures and ICP (Table 3) (14). In a large cohort of 94 moderate to severe TBI patients, post-traumatic seizures (often non-convulsive) were observed in more than one in five patients during the 1st week after injury, despite prophylactic use of phenytoin. The average ICP did not differ between seizure and non-seizure days (13.9 ± 14 vs. 13.5 ± 17 mm Hg; *t*-test, *p* < 0.65). ICP values were also analyzed as serial trends before, during, and after seizures; although the statistical test was not specified. No seizure-related changes in ICP or CPP were observed. However, mean CPP was higher on seizure days (86.6 ± 16.7 vs. 84.3 ± 19.1 mm Hg; t-test, *p* < 0.04). Lastly, ICP and CPP were compared between the seizure and non-seizure groups. The incidence of increased ICP (>20 mm Hg) did not differ between groups (42% seizure vs. 38% non-seizure). Mean ICP was lower in the seizure group than in the non-seizure group (11.8 ± 7.3 vs. 15.6 ± 9.9 mm Hg; two-tailed *t*-test, *p* < 0.001). CPP was higher in the seizure group than in the non-seizure group (85.1 ± 14.5 vs. 83.6 ± 14.5 mm Hg; *t-*test, *p* < 0.03).

**Table 3:** Study characteristics.

Article	Objective	EEG and ICP	Population
Vespa et al. (14)	Incidence of (non-) convulsive seizures and ICP	• Continuous EEG • 12 subcutaneous needle electrodes (10–20) • Mean monitoring time 7.5 ± 4 days • Total 13,659 h of EEG-ICP • EVD	• Moderate severe TBI (GCS ≤ 12); *n* = 94 (78 male; 18–78 years) • Mean initial GCS was 7.1 ± 4.1 and mean Injury Severity Scale was 30.4 ± 9 • Initial GCS score did not differ significantly between the seizure (7.0 ± 3.96) and non-seizure (7.1 ± 4.1) group • A total of 21 patients developed seizures, identified either electroencephalographically or clinically, of whom six experienced status epilepticus (SE). All patients with SE had an increased incidence of hypoxia and died • Phenytoin levels did not differ significantly between (non-)seizure groups • CT-characteristics (number of patients): contusions and SAH and SDH (33), SAH and edema (19), focal contusion (16), SDH (15), EDH (nine), gunshot, bullet retained (two) • A total of 37 patients underwent evacuation of mass lesions < 48 h post-injury. Early evacuation of mass lesions was associated with a decreased incidence of seizures
McNamara et al. (15)	Acute ICP increase and subclinical seizures	• Quantitative EEG (30-min) • 16-channel scalp EEG • Electrode placement not reported • Total monitoring time 14 h • Intraparenchymal ICP probe	• Neurological ICU patients, *n* = 85 • Increase of ICP (>18.4 mm Hg) despite standardized management, *n* = 17 (11 male; 3–66 years) • Nine adult TBI patients, two SAH patients, one SST patient, one ABI • Four pediatric TBI patients • Two adult TBI patients and one pediatric TBI patient had a burst suppressive EEG
Vespa et al. (16)	(Non-) convulsive post-traumatic seizures and ICP	• Continuous EEG • 14-channel (10–20) • 12 subcutaneous needle electrodes • EVD	• Moderate and severe TBI (GCS 3–13); *n* = 20 (10 seizure vs.10 matched non-seizure patients), mean ages 49.7 ± 16 vs. 49.2 ± 16 years • Initial GCS scores of 7.9 ± 3.1 (seizure) vs. 7.6 ± 1.8 (non-seizure) • CT-characteristics (seizure vs. non-seizure, patients): • Contusion: six vs. five; contusion and SDH: two vs. three; Contusion and DAI: two vs. two • Both groups were on average within the therapeutic range for phenytoin, although the seizure group had a higher mean level compared to the non-seizure group (16.7 ± 9.3 vs. 11.1 ± 3.4 mg/dL)
Sanz-García et al. (17)	Directional relationship EEG-ICP	• Continuous EEG • 19 scalp electrodes (10–20) • Mean monitoring time 5.2 ± 2.3 days • Total of 1,055 h of EEG-ICP • Intraparenchymal ICP probe	• Neurological adult ICU patients, *n* = 21 • TBI (15): 11 male; mean age 50 ± 19 (20–81) years • SAH (6): all female; mean age 54 ± 16 (26–76) years • Initial GCS scores were often reported as ranges. The calculated average mean GCS score on admission for all 21 patients was therefore 8.5, with the 15 TBI patients averaging 8.3 and the 6 SAH patients 8.8

ABI, anoxic brain injury; CT, computed tomography, DAI, diffuse axonal injury; EDH, epidural hematoma; EEG, electroencephalography; EVD, external ventricular drain; ICP, intracranial pressure; ICU, intensive care unit; SAH, subarachnoid hemorrhage; SDH, subdural hematoma; SST, sagittal sinus thrombosis.

McNamara et al. (15) prospectively evaluated 85 various neurological ICU patients with ICP-monitoring, to determine if acute increases in ICP were accompanied by subclinical seizures (15). A total of 17 patients had at least one acute episode of raised ICP despite standardized treatment and were included in the analysis. This group consisted of nine adult TBI patients receiving different drug treatments, two of whom had a pharmacologically induced burst-suppression pattern on EEG. No spikes, sharp waves, or electrographic seizures were observed in the EEG of these patients. Seven of the 17 patients experienced further ICP peaks; a definition of an ICP peak was not provided. EEG showed no alterations during these ICP peaks. Topographical spectral analysis was performed before, during, and after the peaks; the quantitative EEG showed no changes.

In 2007 Vespa et al. investigated the relationship between post-traumatic seizures and ICP (16). In a small (*n* = 10) moderate to severe TBI cohort, patients with (all non-convulsive) seizures were compared with a matched cohort without seizures. All patients received phenytoin for at least 7 days. The mean seizure onset occurred 85 ± 7 h after injury. However, seizure timing showed a bimodal distribution. Peaks occurred at 29 ± 14 h and 140 ± 15 h post-injury. Increased ICP was observed during both seizure peaks (29 ± 14 h and 140 ± 15 h). Furthermore, ICP was measured during the 12 h preceding seizures (interictal ICP) and the 12 h following seizure onset (ictal ICP). Mean ictal ICP was higher than interictal ICP (22.4 ± 7 vs. 9.6 ± 5 mm Hg; *p* < 0.002), and ICP remained elevated (>20 mm Hg) for longer in the ictal period (53 ± 33% vs. 7.5 ± 11%; *p* < 0.001). The authors also examined whether seizure type or duration correlated with the magnitude of ICP elevation, but no correlation was found. Patients with status epilepticus and those with intermittent seizures showed similar ICP increases (12 ± 4.5 vs. 13 ± 4.3 mm Hg; *p* > 0.7).

Lastly, the authors compared ICP between seizure and non-seizure groups. During the first 168 h post-injury, mean ICP was higher in the seizure group (17.6 ± 6.5 vs. 12.2 ± 4.2 mm Hg; *p* < 0.001). The seizure group spent more time with ICP > 20 mm Hg (32 ± 28% vs. 6 ± 8.4%; *p* < 0.02). After 100 h post-injury, the seizure group showed a secondary rise in mean ICP, which was not seen in the non-seizure group. Time with ICP > 20 mm Hg also remained higher in the seizure group after 100 h (27 ± 27% vs. 5 ± 8%; *p* < 0.04).

### 3.2 ICP and EEG background patterns

Sanz-García et al. (17) evaluated the directional relationship between EEG activity and ICP in 21 adult ICU patients, of which 15 had TBI and six had subarachnoid hemorrhage (SAH) (17). A total of 1,055 h of continuous EEG-ICP recordings were divided into temporal windows of 5 s to balance EEG signal stability and maintaining adequate temporal resolution to analyze ICP dynamics. For these windows, typical EEG spectral measurements were calculated: spectral entropy (i.e., energy in a specific frequency band) and relative power of the frequency bands: rDelta (>0.5 Hz and < 4 Hz), rTheta (4–7 Hz), rAlpha (7–14 Hz). The linear “Granger causality” (GS) method was used to quantify the strength (the potential dependence) and direction of the relationship between time series of two signals. This method attempts to capture whether values of the first value can be predicted by previous values of the second variable or the other way around. In other words, whether EEG signals can predict ICP or vice versa. Only the percentage of time with increase of ICP (>20 mm Hg) were noted, and EEG activity during these periods was not specifically examined.

In 20 patients, EEG to ICP (i.e., EEG signal predicting ICP signal) showed a higher percentage time of significant Granger causality (GC). In three of these patients (two with TBI and one with SAH), this relationship was fairly weak or non-existent at all. In one (TBI) patient the direction of information was reversed, from ICP to EEG. For EEG to ICP, the mean proportion of time with significant GC was 38%, and eight patients showed significant GC for more than 50% of the time. The highest percentages of time with significant GC occurred in rAlpha range (12 patients), rDelta range (seven patients), and rTheta range (two patients), with characteristic lags of 25–50 s.

Lastly, the influence of drugs effects on the EEG to ICP relationship was examined. The mean percentage of time with significant GC was 39 and 38%, respectively during periods without and with sedative bolus administration. During constant intravenous sedation, the mean percentage of time with significant GC was 38% overall, 39% without bolus, and 38% with bolus only.

## 4 Discussion

We have conducted this systematic review according to the PRSIMA-guidelines and evaluated the relationship between EEG changes and ICP in adult patients with moderate and severe TBI admitted to the ICU. Only four studies fulfilled our selection criteria, three of which had relatively small sample sizes. Three prospective cohort studies, all conducted before 2010, with two originating from the same research group, specifically reported on the incidence of post-traumatic seizures and its association to ICP (14–16). Yet, this relationship remains uncertain. One recent prospective cohort study of predominantly TBI patients showed mainly a directional relationship from EEG to ICP, possibly reflecting neurovascular coupling (17). No specific EEG-ICP analysis during intracranial hypertension was performed in this study. Altogether, our findings implicate that a better understanding of the relationship between intracranial hypertension and cortical activity is necessary in order to develop future multimodal targeted treatment strategies that include personalized EEG-ICP-thresholds.

Patients with severe TBI are admitted to the ICU and treated according to international guidelines with invasive ICP-monitoring which primarily focuses on preventing and treating raised ICP. ICP-monitoring is supported by Class II studies, providing direct moderate-quality evidence, but the overall strength of this evidence base is considered low (3). There is a clear need for integrated noninvasive monitoring techniques to improve our understanding of delayed secondary injury in severe TBI, enabling individualized therapy for early detection and treatment of secondary deterioration to improve outcome (2, 4, 5). A study of two non-traumatic neurocritical patients with different types of cerebral herniation showed that EEG changes were detectable 1 h before clinical deterioration, including a progressive increment of low-frequency power and corresponding asymmetry on the spectrogram (9). In another cohort of seven non-traumatic neurocritical patients with varying conditions, it was demonstrated that transient episodes of presumed elevated ICP, as well as more prolonged and progressive intracranial hypertension, are preceded by nonepileptic EEG changes such as varying degrees of slowing and attenuation (10). These secondary effects can alter cerebral autoregulation, disrupting neurogenic control as vasomotor, chemical, and metabolic, that regulate CBF (8). The relationship between ICP and cortical electrical activity has not yet been systematically reviewed in adult patients with TBI.

In Vespa et al. (14) one in five patients showed post-traumatic seizures during the 1st week after injury despite prophylactic anti-seizure medication (14). Counterintuitively, in the non-seizure group the mean overall ICP was slightly higher with lower CPP. Frequencies of raised ICP were similar between groups, but higher CPP was observed on the day of seizures. No direct seizure-related changes in ICP and CPP were observed. No clear explanation was given whether mean ICP was higher in the non-seizure group, except that is plausible that neurochemical and metabolic changes are interrelated to seizure activity. This data was retrospectively derived with no reliable reporting on short-duration changes and can therefore only be used as indication for general dynamic trends. While there was no association with initial GCS score, early evacuation of mass lesions was associated with a lower incidence of seizures. A sub-group analysis indicated that status epilepticus was associated with higher mortality rates, possibly due to a higher incidence of early hypoxia in this group contributing to seizure development. Overall, post-traumatic seizures may influence ICP, but no conclusive results could be established.

In McNamara et al. (15) quantitative EEG did not show direct alterations of the background pattern, spikes-/sharp waves, or electrographic seizures during- and after ICP peaks in all cases (15). Altogether, this study did not find an association between electrographic changes and ICP, although continuous EEG-monitoring was not used and therefore activity patterns related to a delayed rise of ICP due to loss of autoregulation could have been missed.

Vespa et al. found, in contrast to results of their earlier study (14), that mean ICP was higher in the seizure group, despite higher phenytoin levels (that still were within therapeutic range). Periods of raised ICP were more frequently observed in the seizure group, with a marked bimodal distribution of early- and late (>100 h post-injury) increase of ICP. Within-subject analysis demonstrated that ictal mean ICP was higher than the interictal mean, with also more frequent observations of raised ICP during the ictal period. However, the type and/or duration of seizures, like status epilepticus, were not correlated with the magnitude of ICP. To our knowledge, this study was one of the first showing that seizures are closely associated with episodic increase of ICP and suggest that ICP increases in response to seizures and not vice versa. Is has been hypothesized that this increase of ICP results either from increased CBF due to seizure activity or seizure-related worsening of extracellular cerebral edema which subsequently leads to elevated ICP.

Sanz-García et al. (17) evaluated the directional relationship between EEG background patterns and ICP in 21 adult ICU patients, consisting of 15 TBI and six SAH patients (17). Across all patients, the recordings showed an average of 38% significant GC from EEG to ICP. This indicates that EEG features tended to precede ICP changes, with characteristic lags of 25–50 s, mostly in the rAlpha range. In three patients (two with TBI and one with SAH), this directional relationship was limited or absent, and in one TBI patient the directionality was reversed, with ICP changes preceding EEG activity. These findings suggest that, in most patients, EEG activity precedes ICP dynamics in a manner consistent with neurovascular coupling; linking cortical activity to vascular responses. However, this study only mentioned the percentage of time of increased ICP and did not specifically evaluate EEG changes during intracranial hypertension, for which the pathophysiology may differ. During these circumstances it can be proposed that cortical activity is affected following increase of ICP, respectively due to decrease of CPP and CBF, which will diminish oxygen and metabolic demands. No differences in GC values (37%−39%) on EEG-ICP were observed between subsets of drug treatment groups. It has been hypothesized that the absence of an effect of several anesthetic agents on total GC may indicate that, during periods without detectable GC, these agents suppress or interfere with neurovascular coupling, thereby uncoupling EEG activity from ICP fluctuations.

## 5 Conclusion

In conclusion, in this systematic review we show that in patients with TBI and ICP-monitoring the relationship between post-traumatic seizures and increase of ICP remains uncertain. There are indications that neuronal activity precedes ICP, however no specific EEG analysis during intracranial hypertension was performed in this study. Future well-powered studies integrating multimodal monitoring are required to further elucidate the EEG-ICP relationship, particularly to develop personalized strategies incorporating EEG-ICP-thresholds for early targeted treatment.
